# Ameliorative effect of diazepam against ethanol-induced mitochondrial disruption in brains of the mice

**DOI:** 10.1016/j.toxrep.2023.10.014

**Published:** 2023-10-24

**Authors:** Seyed Khosro Ghasempouri, Zahra Askari, Hamidreza Mohammadi

**Affiliations:** a Department of Emergency Medicine, School of Medicine, Antimicrobial Resistance Research Center, Ghaem Shahr Razi Hospital, Mazandaran University of Medical Sciences, Sari, Iran; b Department of Toxicology and Pharmacology, Faculty of Pharmacy, Mazandaran University of Medical Sciences, Sari, Iran; c Pharmacutical Sciences Research Center, Hemoglobinopathy Institute, Mazandaran University of Medical Sciences, Sari, Iran

**Keywords:** Ethanol, Oxidative stress, Mitochondria, Diazepam

## Abstract

Brain oxidative damage and neurodegeneration by ethanol (ETH) are considered as important factors that triggered by oxidative stress. Recently, the abuse of diazepam (DZM) has increased by alcoholism-addicted patients. The present study evaluated the effects of combination treatment of ETH with DZM on oxidative damage induced in brain mitochondria of the mice. Only ETH (0.3, 0.6, and 2.5 g / kg) and ETH+ DZM (2.5 mg / kg) were administered intraperitoneally (ip) to the mice. Pathological changes and oxidative stress biomarkers including ROS, lipid peroxidation, carbonyl protein, mitochondrial function, and glutathione content were evaluated in brain mitochondria after 42 days. Results indicated that co-treatment of DZM and ETH significantly reduced mitochondrial toxicity, oxidative damage, pathological changes and increased level of glutathione. Subchronic ETH administration induced brain oxidative damage, mitochondrial disruption, and serious damage to the brain cells. Whereas, combination treatment improved oxidative damage, mitochondrial function, and pathological changes in brain cells after intoxication by ETH. These findings suggest antioxidant effect of DZM in combination with ETH and can be considered in reducing oxidative stress and mitochondrial damage attenuation in the brain. Combination therapy may be a better therapeutic candidate for prevention of brain oxidative damage induced by ETH.

## Highlights

• Ethanol induce cardiomyocytes mitochondrial toxicity.
• Oxidative stress biomarkers and mitochondrial function are disrupted by Ethanol.
• Diazepam attenuates oxidative damage in heart mitochondria after Ethanol induce toxicity.

## Graphical abstract

**Figure:**
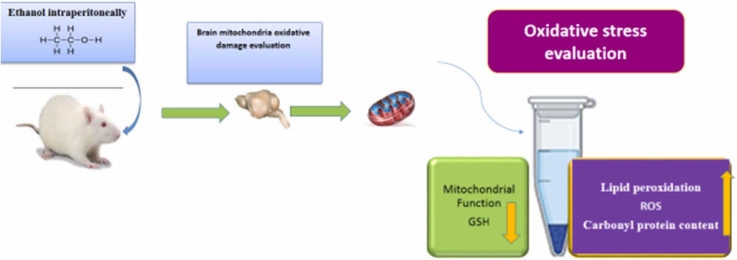

## 1 Introduction

Ethanol (ETH) is known as an addictive compound, and alcoholism-addicted people suffer from many debilitating diseases such as fatty liver, cirrhosis, psychical addiction, hypertension and over than one hundred disabling diseases. Complex mechanism of action and various toxic effects in different organs including liver, heart, brain, pancreas kidney, and skeletal muscle have been reported as a result of ETH consumption [1], [2]. It has been stated that, ETH consumption increases pro-inflammation cytokines (Interleukin-1; IL1, IL6, and tumor necrosis factor; TNFα) and induces inflammatory reactions in the brain that can improve to nervous degeneration [3], [4]. In addition, ETH can decrease cognitive ability, disturb behavioral constancy, consequently leading to emergence of antisocial behavior [5].

Many conducted studies demonstrated that ETH induced oxidative stress [5], [6]. When the body is exposed to the oxidative stress inducers, such as ETH, the discipline and equilibrium of vital systems is disturbed and consequence that disabling diseases such as neurodegenerative disease, diabetic disease, and malignant cancers can be revealed [1], [3], [6]. Oxidative stress triggers reactions spread a large amount of Reactive Oxygen Species (ROS) in the body. ROS formation causes basic oxidative damages in the DNA of cell nucleus and even influences on gene transcription. Moreover, it increases expression of transcription factors such as NFĸB and AP1 contributing in transcription of inflammatory cytokines (IL1, 6, and TNFα). It has been stated that ROS induce apoptosis by releasing mitochondrial cytochrome C, thereby causing development of many diseases especially neurodegenerative disorders [7]. It has been found that, mitochondria are main facilitator of ETH toxicity in which mitochondrial alterations are associated with severity of ETH consumption. Neuronal death and brain cellular damage has been reported as a consequence of ETH intoxication [4]. Nevertheless, ETH can impair signaling of neurotransmitters at the cellular level [8]. Moreover, ETH stimulates ROS formation [9] and motivates inflammatory processes. Eventually, these procedures could be responsible for damage induced by ETH in the brain [10]. Mitochondria are basic source of ROS formation in the brain cells, and they are essentially influenced by oxidative damage prompted by ETH toxicity [11]. Moreover, mitochondrial dysfunction plays an essential role in enhancement of pro-inflammatory processes [12]. Finally, it has been demonstrated that, ETH enhances ROS generation, impairs ATP formation [13], [14], impairs mitochondrial respiration [15], and ultimately induces brain cell death by opening Mitochondrial Permeability Transition Pore (MPTP) [10], [16].

Diazepam (DZM) is one of the best known benzodiazepine drug, and is used for treatment of neurological and psychiatric diseases with protective effect on the nervous system [17], [18]. It has been demonstrated that, DZM increases affinity of γ-Aminobutyric acid type A (GABA_A_) receptors, and through which prevents induced adverse effects. DZM is known as a GABA allosteric activator and facilitates hyperpolarization process in neuron cells. Protective effect of diazepam on the central nervous system is considered as a subject of interest for the neurologists [17], [19], [20], [21], [22].

Benzodiazepine drugs are used in Alcohol Withdrawal Syndrome (AWS) for decreasing severity of AWS symptoms. DZM is mainly administered as the first line drug in treatment of AWS. ETH increases the brain's inhibitory systems through GABA_A_ receptor, and chronic exposure to alcohol leads to down-regulation of GABA_A_ receptors and up-regulation of NMDA receptors [23]. DZM and other benzodiazepine drugs activate GABA_A_ ion channel and so increase GABA neurotransmitter which in turn decreases AWS symptoms [24], [25], [26], [27].

Recently, the abuse of DZM has increased by alcoholism-addicted patients. Some alcoholism-addicted people consume DZM along with ETH to intensify their sense of calmness and experience more euphoria effects. Accordingly, the present study was conducted to investigate the effects of simultaneous consumption of DZM and ETH on oxidative damage induced in brain mitochondria in BALB/c male mice.

## 2 Material and method

### 2.1 Materials

All the chemical substances used in this study were prepared from the Merck (Darmstadt, Germany) and Sigma Chemical Co. (St. Louis, MO, USA). Diazepam from Chemidarou Industrial Company. Iran, were purchased.

### 2.2 Experimental design

In this subchronic (6 weeks) study, 54 BALB/c male mice (20–25 g; 6–8 weeks old) were used. Animals were retained in a light-controlled room (12-h day/night cycle) and were allowed free access to food and water. All the experiments were done in accordance with the recommendations on animal experiments by the ethics committee of the Mazanderan University of Medical Sciences (MAZUMS; Animal Ethical Committee with the research ethic certificate number: IR.MAZUMS.REC.1396.3033).

Animals were injected intraperitoneal (Ip) for 6 weeks in 9 groups including different doses of ETH (0.3, 0.6, 1.2, 2.5, and 5 g / kg) + DZM (2.5 mg/kg), and only ETH (0.3, 0.6, and 2.5 g/kg). The normal saline group was considered as a control group. Animals were anesthetized with ketamine/xylazine (7/1) and then sacrificed after 6 weeks and oxidative stress biomarkers including; ROS formation, lipid peroxidation (LPO), protein carbonyl (PC) content, mitochondrial function and glutathione (GSH) content were measured in brain isolated mitochondria.

### 2.3 Brain mitochondrial preparation

Mice were sacrificed after 24 h of the last injections and the mice brains were brought out immediately and were washed with mannitol buffer. Then pieces of brain were homogenized and mitochondria were isolated by different centrifugation (2000g for 10 min; for first time and the supernatant was separated carefully and was centrifuged at 11000 g for 10 min in 4 ^º^C) and vital mitochondria were collected. Sediments of mitochondria, were dispersed in tris buffer for assessment of GSH, PC, LPO and mitochondrial function experiments. Some of the sediment was dispersed in breathing buffer for measurement of ROS test [28], [29].

### 2.4 Measurement of LPO content

0.3 ml of thiobarbitoric acid (TBA) was added to mitochondrial fractions in all the tubes and was placed in a boiling water bath for 30 min. After that, all the tubes were placed to an ice-bath, and 4 ml of n-butanol was added to each tube. Finally, samples were centrifuged at 1000 g for 10 min and the amount of the Malondialdehyde (MDA) content was measured by spectrophotometric method [30].

### 2.5 Measurement of PC

80 µl of trichloroacetic acid (TCA) was added to 1 ml of mitochondrial fraction and was placed in 4 ºC for 15 min. 30 µl of 2, 4 dinitro phenyl hydrazine (DNPH) was added to each sample. All the sample tubes were incubated at room temperature for 1 h. All of samples were washed three times with ethanol/ethyl acetate (1: 1 v/v), and at the end, 400 µl guanidine hydrochloride (6 M) were added and the PC content was measured by reading the absorbance at the 365 nm wavelength by ELIZA reader (Tecan, Rainbow Thermo, Austria) [28], [29].

### 2.6 Measurement of GSH content

250 µl of TCA (10%), 250 µl of EDTA, 2 ml phosphate buffer and 200 µl DTNB were added to 1 ml of mitochondrial fractions in the all tubes and all of samples were centrifuged at 7000 g for 10 min. The GSH content was measured by spectrophotometer (UV-1601 PC, Shimadzu, Japan) at 412 nm and expressed as μM [29].

### 2.7 Measurement of ROS in brain mitochondria

Isolated mitochondria were measured by fluorescence spectrophotometer. Briefly, 10 µl of dicholorofluorescin diacetat (DCFH-DA) and 2 ml of tries buffer were added to 1 ml of mitochondria fractions (1 mg protein/ml) and were incubated 15 min at 37 °C. The amount of ROS formation was measured at the excitation and emission (485 and 520 nm) wavelength [29].

### 2.8 Determination of mitochondrial function (MTT assay)

Mitochondrial function was assessed by measuring the reduction of MTT (3-[4, 5-dimethylthiazol-2-yl]−2,5-diphenyltetrazolium bromide by mitochondria [31]. Briefly, 40 µl of MTT solution was added to 1 ml mitochondrial sample and all the tubes were incubated at 37 ^⸰^C for 30 min. All the tubes were centrifuged at 7000 g to 10 min. At the end, the 1 ml of Dimetylsolfoxide (DMSO) was added to bottom sediment and the absorbance was calculated at 580 nm.

### 2.9 Measurement of protein concentration

Protein content was assessed by using the Coomassie blue protein-binding method [32]. Bovine serum albumin was used in this test as the standard for determination of protein content.

### 2.10 Histopathological examination

The mice brains were separated and fixed in 10% of buffered formalin for at least 24 h. All of the samples were stained with Hematoxylin and Eosin (H&E). The sample slides were examined under the light microscope. Histopathological damages in the brain tissues were evaluated and a score between severe = ++ +, moderate = ++ , mild = +, and normal histology were dedicated to range of damage.

After the separation of samples, the brains of the mice were fixed in 10% buffered formalin for 24 h. After dehydrating with alcohol and clarifying with xylene, samples were molded with paraffin. Then, 5-micron sections were stained with Hematoxylin and Eosin (H&E). A histologist blinded to the treatment groups examined sample slides by light microscopy. Brain tissues were evaluated regarding tissue degeneration, hemorrhage, vacuolation, necrosis, and pyknosis of neurons. Accordingly, the score + ++ was assigned to severe damage, + + to moderate damage, and + to mild damage tissue structure.

### 2.11 Statistical Analysis

Data were described as mean±SD and results were analyzed using Graphpad Prism software, Ver 6. ANOVA followed by Tukey test were implemented to compare the means. *P* values less than 0.05 were considered as significant**.**

## 3 Results

**Fig. 1:**
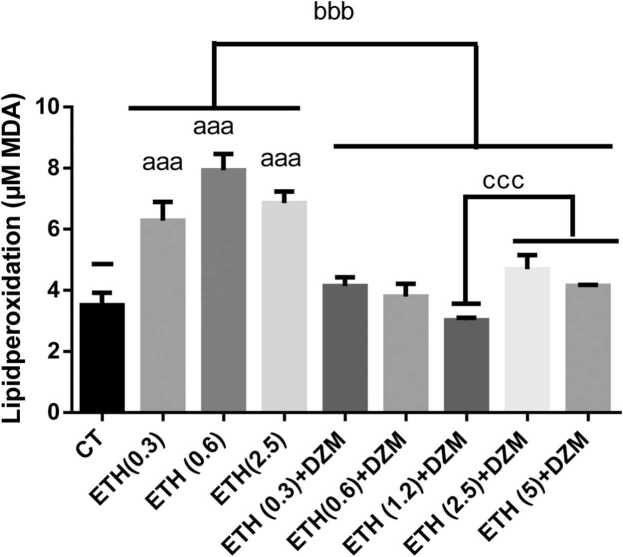
Effects of combination of Diazepam (DZM; 2.5 mg/kg) and Ethanol (ETH; with different doses: 0.3, 0.6, 1.2, 2.5, and 5 g/kg) on lipid peroxidation in mice brain mitochondria after 6 weeks treatment. Values represented as mean ± SD (n = 6). ^aaa^(p < 0.001): Significantly different from control group (CT). ^bbb^(p < 0.001): significantly different from ETH (0.3,0.6, and 2.5 g/kg) groups. ^ccc^(p < 0.001): significantly different from ETH (1.2 g/kg) +DZM.

In this study, the effect of simultaneous use of DZM and ETH on brain mitochondria oxidative damage showed that ETH (at all doses) significantly (p < 0.001) increased LPO compared to control group (Fig. 1). Co-retreated mice with DZM (ETH+DZM) significantly (p < 0.001) decreased LPO in mice brain mitochondria when compared with ETH groups. LPO significantly (p < 0.001) decreased in ETH (1.2) +DZM group compared to ETH (2.5 and 5) +DZM groups (Fig. 1).

**Fig. 2:**
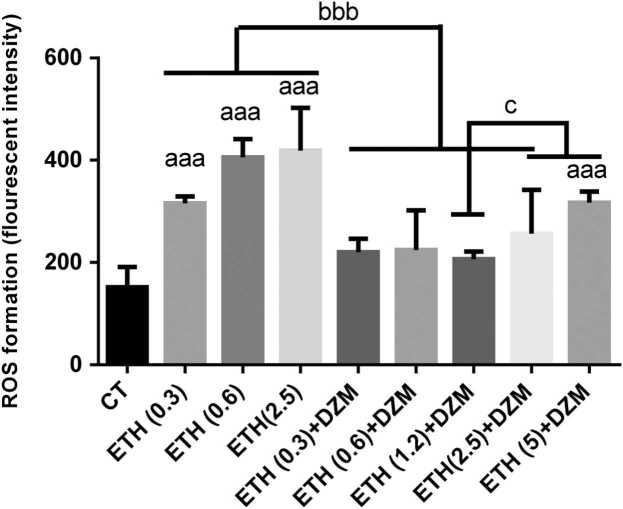
Effect of combination of Diazepam (DZM; 2.5 mg/kg) and Ethanol (ETH; with different doses: 0.3, 0.6, 1.2, 2.5, and 5 g/kg) on ROS formation in mice brain mitochondria after 6 weeks treatment. Values represented as mean ± SD (n = 6). ^aaa^(p < 0.001): Significantly different from control group (CT). ^bbb^(p < 0.001): significantly different from ETH (0.3,0.6, and 2.5 g/kg) groups. ^c^(p < 0.05): significantly different from ETH (1.2 g/kg) +DZM.

ROS generation in mice brains mitochondria after different treatments with ETH and DZM showed that the levels of ROS were significantly increased (p < 0.001) in ETH treated groups (0.3, 0.6, and 2.5 g/kg) and ETH (5) +DZM groups compared to the CT group. ROS levels significantly were decreased in mice treated with DZM (ETH+DZM; 0.3, 0.6, 1.2, and 2.5 g/kg) groups when compared to all ETH doses (0.3, 0.6, and 2.5 g/kg) groups (Fig. 2). Also, a significant decrease (p < 0.001) in ROS formation was observed in ETH (1.2) +DZM group compared to high doses of ETH (2.5 and 5) +DZM groups (Fig. 2).

**Fig. 3:**
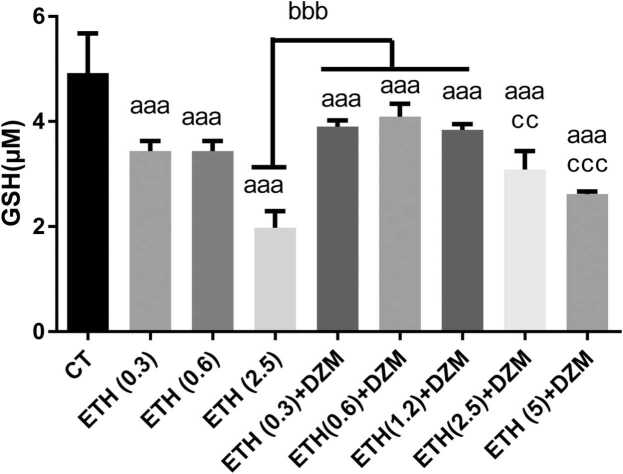
Effect of co-treatment of Diazepam (DZM; 2.5 mg/kg) and Ethanol (ETH; with different doses: 0.3, 0.6, 1.2, 2.5, and 5 g/kg) on glutathione content in mice brain mitochondria after 6 weeks treatment. Values represented as mean ± SD (n = 6). ^aaa^(p < 0.001): Significantly different from control group (CT). ^bbb^(p < 0.001): significantly different from ETH (2.5 g/kg) group. ^ccc^(p < 0.001), ^cc^(p < 0.01): significantly different from ETH (0.3,0.6, and 1.2 g/kg) +DZM groups.

GSH contents were significantly decreased (p < 0.001) in ETH treated groups at all doses (0.3, 0.6, and 2.5 g/kg) and all co-treated groups (ETH+DZM (0.3, 0.6, 1.2, 2.5, and 5 g/kg) when compared with the CT group (Fig. 3). GSH content in mitochondria significantly decreased in ETH at dose of 2.5 g/kg compared to ETH+DZM (0.3 and 0.6 g/kg) groups.

**Fig. 4:**
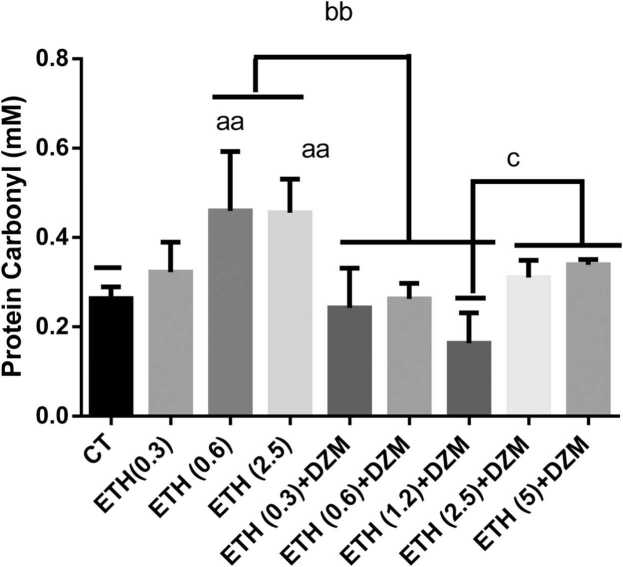
Effect of co-treatment of Diazepam (DZM; 2.5 mg/kg) and Ethanol (ETH; with different doses: 0.3, 0.6, 1.2, 2.5, and 5 g/kg) on protein carbonyl content in mice brain mitochondria after 6 weeks treatment. Values represented as mean ± SD (n = 6). ^aa^(p < 0.01): Significantly different from control group (CT). ^bb^(p < 0.001): significantly different from ETH (0.6 and 2.5 g/kg) group. ^c^(p < 0.05): significantly different from ETH (1.2 g/kg) +DZM groups.

PC as a marker for evaluation of proteins oxidation was assessed in this study. The level of PC in the brains mitochondria of male mice significantly (p < 0.01) increased in ETH (0.6 and 2.5 g/kg) groups compared to the CT group, whereas it was diminished significantly in ETH+DZM (0.3, 0.6, and 1.2 g/kg) groups when compared with ETH (0.6 and 2.5 g/kg) groups. Also, PC in ETH+DZM (2.5 and 5 g/kg) groups were significantly increased (P < 0.05) in comparison with ETH 1.2 g/kg +DZM group (Fig. 4).

**Fig. 5:**
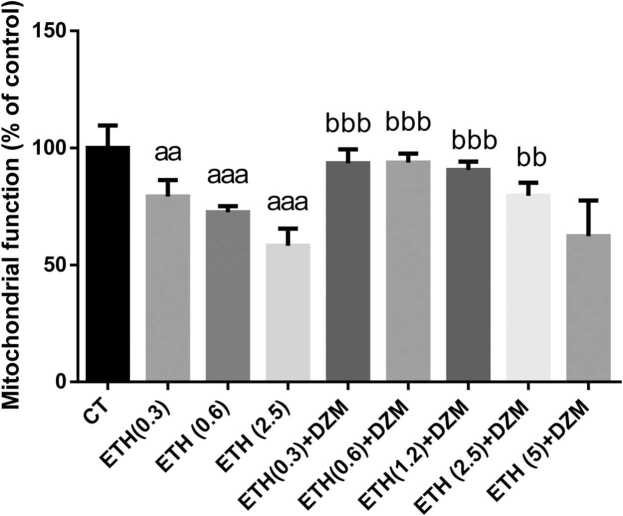
Effect of co-treatment of Diazepam (DZM; 2.5 mg/kg) and Ethanol (ETH; with different doses: 0.3, 0.6, 1.2, 2.5, and 5 g/kg) on mitochondrial function in mice brain cells after 6 weeks treatment. Values represented as mean ± SD (n = 6). Data are presented as percentage of changes to control group (CT) group. ^aaa^(P < 0.001), ^aa^(p < 0.01): significantly different from control group(CT). ^bbb^(p < 0.001), ^bb^(P < 0.01): significantly different from ETH 2.5 g/kg.

MTT assay test was used for evaluation of mitochondrial function in mice brains after treatment with ETH and/or ETH+DZM. As shown in Fig. 5, mitochondrial function significantly decreased in all ETH treated groups (0.3, 0.6, and 2.5; 20.67%, 27.51%, and 41.58% respectively) in comparison with CT group. Whereas, it significantly increased by DZM in co-treated groups of ETH+DZM (0.3, 0.6, 1.2, and 2.5 g/kg; 35.33%, 35.56%, 32.51%, and 21.46% respectively) when compared with ETH 2.5 g/kg group.

### 3.1 Histopathological findings in the brain of mice

**Fig. 6:**
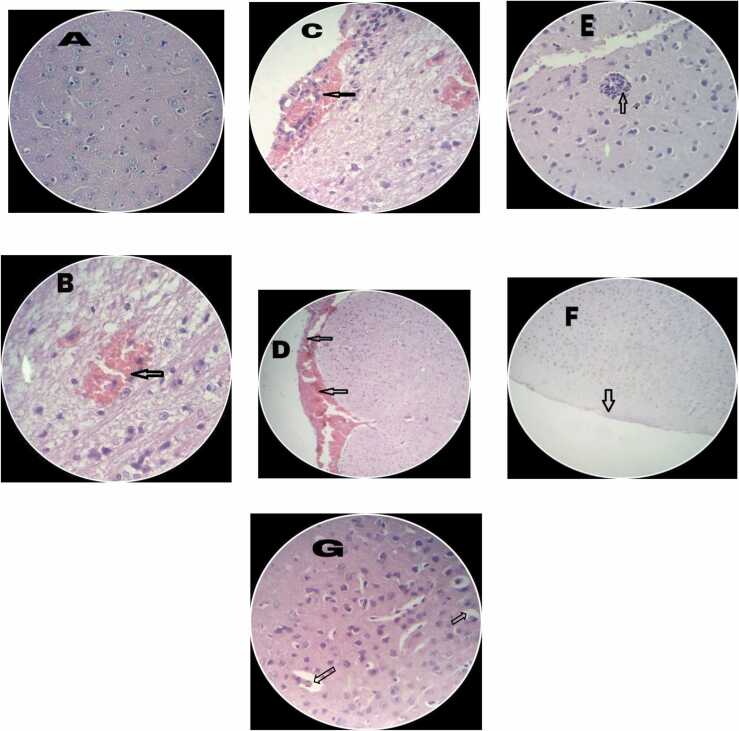
Histopathological changes of co-treatment of Diazepam (DZM; 2.5 mg/kg) and Ethanol (ETH; with different doses: 0.3, 0.6, 1.2, and 2.5) on brain tissues. (A) the control group that is shown a normal structure (H&E staining; 400 ×magnification). (B and C) the microscopic section of brain tissues of mice that have received only 2.5 g/kg ETH which indicates hemorrhage (H&E staining; 400 ×magnification). (D) the 2.5 g/kg ETH group that is shown a hemorrhage under the pia matter (H&E; 100 ×magnification). (E) 2.5 g/kg ETH group that shown lymphocytes and plasma cell aggregation (H&E; 400 ×magnification). (F) The 2.5 g/kg ETH +DZM group (H&E; 100 ×magnification). (G) The 2.5 g/kg ETH + DZM which indicates perivascular edema and cell swelling (H&E; 400 ×magnification).

Histopathological changes of brain tissues are presented in Table 1 and Fig. 6 (A-G). All histological findings were normal in the brain of control group. According to figure B-D, the group had received only 2.5 g/kg ETH are shown cell swelling in cortical layer of cerebrum with mild perivascular lymphoplasmocytic infiltration and congestion and perivascular edema especially under piamater in comparison with control group (Figure A and Table 1). Also, lymphocytes and plasma cells aggregation were shown in the group that received only 2.5 g/kg ETH (Figure E and Table 1). According to the figure F and G, the group that received 2.5 g/kg ETH+DZM, the level of perivascular edema, congestion and cell swelling significantly decreased when compared with only 2.5 g/kg ETH group.

**Table 1:** Effect of diazepam (DZM) 2.5 mg/kg and Ethanol (ETH) with different doses (0.3, 0.6, 1.2, and 2.5 g/kg) on histopathological changes in brain tissues of mice after 6 weeks treatment (n = 6). Histopathological criteria were determined semi-quantitatively from mild (+) to moderate (++) and severe (+++).

**Groups**	**Brain**
**Control**	Normal
**ETH 0.3 g/kg**	Normal
**ETH 0.6 g/kg**	Normal
**ETH 2.5 g/kg**	Moderate congestion (++), moderate prevascular lymphoplasmocytic infiltration (++) and mild prevascular edema (+)
**ETH 0.3 g/kg+ DZM 2.5 mg/ kg**	Normal
**ETH 0.6 g/kg+DZM 2.5 mg/kg**	Normal
**ETH 1.2 g/kg+DZM 2.5 mg/kg**	Normal
**ETH 2.5 g/kg+DZM 2.5 mg/kg**	Mild congestion (+)

## 4 Discussion

In this study, the effect of simultaneous consumption of DZM and ETH on oxidative damage induced by ETH was investigated. Results of this study demonstrated that, ETH at different doses (0.3, 0.6, and 2.5 g/kg) induced oxidative damage in brain cell mitochondria. Co-treatment of DZM (2.5 g/kg) and ETH decreased levels of oxidative biomarkers and mitochondrial functional in brain cells of the mice.

ETH is a constant and reliable producer of neuronal toxicity, especially when exposure occurs through periods of increased neuronal vulnerability. Also, neuronal dendritic shrinkage has been recognized in ETH exposure [13]. ETH is a compound with quick effect because of its water and fat soluble properties. ETH is an addictive compound and rapidly metabolized to acetaldehyde and induce direct and indirect toxic effects in different tissues on humans and animals. Microsomal ETH oxidation system (CYP2E1), Catalase, and Alcohol Dehydrogenase (ADH) are main pathways of ETH metabolism contributing in ETH toxicity that leads to formation of ROS, lipid peroxidation, protein oxidation, apoptosis initiation in numerous tissues, and finally resulting in emergence of oxidative damage. Initiation and progression of severe abnormalities in several organs are contributed to oxidative damage induced by ETH [4], [5], [6], [33].

Results of our study showed that, ETH induced brain mitochondria toxicity through increasing brain tissue damage, enhancing of ROS, PC and LPO levels and diminishing GSH content and disturbance in mitochondrial function (Fig. 1, Fig. 2, Fig. 3, Fig. 4, Fig. 5). Whereas, it was confirmed that, DZM co-treatment was able to attenuate all above mentioned abnormalities in brain mitochondria of mice in a 6-week period.

Brain is rich of Polyunsaturated Fatty Acids (PUFAs) causing initiation of oxidative reactions and disturbance in the equilibrium of oxidative stress [34]. Previous studies demonstrated that brain is one of the most important targets damaged by ETH because, Unsaturated Fatty Acids (UFAs) available in the brain can initiate oxidative stress reaction, which in turn can cause functional and structural defects in the brain [35], [36]. The brain is a membrane dense organ and ETH influences various kinds of movements within the membranes leading to the increase in amount of fluid membranes. It has been stated that, lipid milieu and embedded proteins are disturbed by ETH [37]. It has been found that, the ETH increases micropinocytic vesicles and number of mitochondria in the cytoplasm. Moreover, proliferation of smooth endoplasmic reticulum and golgi system is prominent by ETH consumption. Significant edema along with occurrence of abundant mitochondria with abnormal shape and huge size has been reported in astrocytic processes surrounding the vessels by ETH [1]. All of these processes might occur due to an enhanced permeability of the Blood-Brain Barrier (BBB) as a consequence of toxic effects of ETH consumption [1], [38]. Also, enhanced levels of ROS, lipid and protein oxidation consequently resulting in neuronal death have been reported in chronic alcohol abuse [39].

Glutathione is an important antioxidant in tissues capable of preventing oxidative damage produced by ROS. Lipid peroxidation is a biomarker for oxidative damage. Generation of free radicals and presence of oxidative stress causes overproduction of MDA as a result of which GSH content decreases[40]. Our study results revealed that, ETH significantly increased level of MDA and diminished content of GSH in the mitochondria of brain tissues (Fig. 1, Fig. 3). It has been stated that, ETH (40% v/v, 5 g/kg) administration every 12 h reduced GSH and enhanced MDA levels in heart and liver,which is similar to our investigation [33]. Another study revealed that, administration of ETH (40% w/v) 2 g/kg/day for 30 days enhanced LPO and diminished GSH levels confirming our results [41]. It has been demonstrated that, oxidative damage occurs in the male rats treated with ETH (35% w/w) for 30 weeks [42]. Moreover, ETH (50%) had the same effects on MDA and GSH in the liver at the dose of 12 ml/kg for 15 days [43].

In our investigation, DZM (2.5 g/kg/day, 6 weeks) restored oxidative damage induced by ETH. Therefore, it can be concluded that, the DZM (2.5 g/kg) has shown neuroprotective effect against the ETH toxicity at all used doses indicating that DZM has played as an antioxidant and had protective effect against disruption of the brain mitochondria.

It has been stated that, mitochondrial bioenergetic function is strictly compromised following chronic alcohol consumption. Nevertheless, underlying mechanisms responsible for mitochondrial disruption remain unknown.

ETH acts as a stress inducer at cellular level and cause damaging effects on the mitochondria by activation of mitochondrial intrinsic apoptotic pathway [44], [45]. It has been shown that, alteration in regulation of mitochondrial dynamics might be a communal pathogenic pathway of ETH -induced toxicity [46], [47]. Control of mitochondrial dynamic processes is tightly regulated by cellular pathways. Moreover, it has been revealed that, mitochondrial fission is commonly controlled by optic atrophy 1 (OPA1), dynamin-related protein 1 (Drp1), or the pro-apoptotic B-cell lymphoma 2 (Bcl-2) family member Bcl-2-associated X protein (Bax), amongst other proteins [48], [49]. Furthermore, Bax involves Drp1 function in order to be concentrated in the foci alongside the mitochondrial membrane [50], [51]. ETH increases mitochondrial fission through Drp1 mitochondrial translocation and OPA1 proteolytic cleavage in a concentration-dependent manner. Mitochondrial potential disturbances arbitrated by alterations in mitochondrial complex IV protein level as well as enhanced ROS formation has been observed in ARPE-19, a human retinal pigment epithelial cell line. In addition, canonical autophagic pathway, as indicated by autophagosome development and autophagy regulator elements including Autophagy related (ATG5-ATG12), Beclin1, and Phospho-p70 S6 Kinase (P-S6 kinase) has been reported to be activated by ETH. Also, inhibition of autophagy increases mitochondrial fission and cell death [52]. Therefore, mitochondria as main source of ROS is the trigger of autophagy activation. The increase in ROS generation impairs antioxidant system. ROS induce over stimulation of transcription factors such as Activator protein 1 (AP1) and Nuclear factor-κB (NF-ĸB). Also, during dose -dependent process, ROS amplify expression of tumor suppressor gene (p53) and stimulate transcription of pro-apoptotic genes (BH3 interacting-domain; Bid, BCL-2 antagonist killer; Bak, and Bax) thereby activating mitochondrial apoptosis pathway [53]. ETH motivates oxidative damage, both by enhancing ROS generation and by diminishing cellular defense mechanisms. Mitochondrial DNA is an important target of ETH -associated increases in oxidative damage. Damage induced to the mitochondrial DNA by ETH impairs mitochondrial function promoting oxidative stress in the cell, leading to a noxious cycle of accumulating cell damage that is more pronounced with advancing age. Activation of the mitochondrial permeability transition has been proven via abandoned mitochondrial formation of ROS so that, sensitivity of cells is enhanced to other pro-apoptotic or damage signals. Apoptotic and necrotic cell death in response to other benign and beneficial challenges and its contribution to onset or progression of ETH -induced mitochondrial disruption has been reported in a previous study [5], [44], [54].

Our study results indicated, (Fig. 5) mitochondrial function significantly decreased by ETH-induced toxicity (about 20–42%) whereas, mitochondrial function improved by DZM in groups treated with combined intervention (35.33%, 35.56%, 32.51%, and 21.46%, respectively) in comparison with the group treated with ETH at the dose of 2.5 g/kg. It can be concluded that, DZP may contribute in processes of apoptotic and necrotic cell death in response to benign challenges as well as onset or progression of ETH and upon which prevents mitochondrial disruption and protects mitochondrial integrity.

Radical scavengers are compounds removing ROS and decreasing stress oxidative biomarkers. Glutathione, superoxide dismutase (SOD), and catalase are known the most important radical scavengers. It has been reported that, some drugs such as anesthetic drugs act as free radicals scavenger [55]. Numerous studies have been stated that, DZM has neuroprotective effect, especially after ischemic attack and stroke. Previous studies indicated that, DZM has protective effect on neurons in Cornu Ammonis (CA1) hippocampus region and prevents neurodegeneration after ischemic attack. It has been shown that, when cerebral brain is encountered with severe oxygen deficiency, then inhibitory effects of GABA neurotransmitter significantly decrease, as a result of which, severity of brain damage increases [56], [57]. Hence, in this regard, DZM is an attractive drug for the neurologists and neurosurgeons. Despite its auspicious neuroprotective properties, exact neuroprotection mechanism of DZM is not fully understood. It has been stated that, DZM influences on translocator proteins located at the contact site (between inner and outer mitochondrial membrane) separately from other activities such as gamma-amino butyric acid-A receptor stimulation [17]. Mitochondria linked apoptosis is regulate by translocator proteins [58].

Different in vivo and in vitro studies have stated that, neuronal development is changed by ETH [59]. ETH has destructive effect on vital compounds such as protein, and nucleic acid that can cause extensive destructive effect on brain astrocyte [60]. In our study, combination therapy significantly attenuated damage induced in mitochondria, and DZM could attenuate free radicals induced by ETH through modulating of oxidative processes in the brain. Moreover, improvement of mitochondrial function, integrity, and oxidative damage in brain cells was observed with combination therapy (Fig. 1, Fig. 2, Fig. 3, Fig. 4, Fig. 5). As a result, DZM in combination with ETH may be a better therapeutic option in management of ETH toxicity with co-occurring oxidative stress. Thus, DZM influences on mitochondria apart from other reported mechanisms. In our study, DZM potentiated antioxidant capacity of cells in brain of the mice exposed to ETH. DZM has been revealed to modulate mitochondrial function in the brain tissues [17]. Thus, in combination therapy of ETH and DZM, potential mitochondrial-dependent activity may occur. Results of the current study revealed that, level of LPO was higher in the ETH -exposed groups and oxidative damage increased in all the brain regions, which is similar to findings of other researches [5], [6], [35], [37], [38], [54], [61]. Co-treatment with DZM alleviated amount of LPO in brain of the mice exposed to ETH -induced oxidative stress. Nevertheless, this study was the first study reported the effect of DZM on extent of ROS in brain mitochondria in mice.

Furthermore, reports also demonstrated that, antioxidant defense system is mitigated in the brain tissue after administration of ETH [62], [63].

Our findings revealed that, combination of DZM and ETH mitigated reduction of GSH content in the brain mitochondria at all doses of DZM (Fig. 3). Hence, it can be assumed that, combination therapy may prevent glutathione content depletion, improving antioxidant defense system and restoring mitochondrial function and integrity, and mitigating mitochondrial oxidative damage in all the brain regions.

DZM is widely prescribed for treatment of insomnia, anxiety, or seizure disorders. Moreover, in vivo and in vitro studies have shown that, DZM is a potent neuroprotective agent. DZM influences on the CNS by exerting protective effect related to enhancement of GABA neurotransmission via central benzodiazepine (BZD) receptors. Furthermore, it has been stated that, DZM has multi-factor mechanism of action, such as suppression of stress-induced cytochrome c release to cytosol which revealed to have similar neuroprotective effect [17]. Additionally, this influence can be due to direct effect of DZM on mitochondria. Previous findings showed that, DZM influences on translocator protein (TSPO, the mitochondrial benzodiazepine receptor previously named as peripheral benzodiazepine receptor),which is a pharmacologically distinctive type of receptor [64]. TSPO is principally located in the outer membrane of mitochondria and is not associated with GABA_A_ receptor (functionally or structurally). TSPO is essentially found in glial cells in the CNS [65]. Also, TSPO is involved in stress response under neuropathological conditions. Moreover, TSPO contributes in adenine nucleotide transporter, component of the MTP. MTP is a multiprotein complex involved in initiation and regulation of apoptosis [58], [64]. Our results revealed that, good neuroprotection presented by DZM following ETH administration is based on its involvement in prevention of mitochondrial disruption leading to enhanced neuronal oxidative damage inhibition and maintaining of mitochondrial function and integrity of mitochondria.

Neuroradiological investigations have confirmed that, chronic exposure to the ETH causes loss of gray and white matter volumes[39]. Also, in chronic alcoholism, myelin appears to be reduced and cell necrosis occurs [1]. Our histopathological observations exhibited that, ETH could induce cell swelling in cortical layer of cerebrum with mild perivascular lymphoplasmocytic infiltration and congestion and perivascular edema especially under piamater in comparison with control group (Fig. 6A). Our results are compatible with previous studies stated that, ETH induced brain cytotoxic edema, neurodegeneration, and histopathological changes in brain of the rats [66], [67], [68]. Our findings revealed that, combination therapy with DZM significantly decreased level of perivascular edema, congestion, and cell swelling (Figs. 6F and 6G). Structural changes were regressed by DZM administration. The animals treated with ETH in combination with DZM showed marked improvement with almost normal morphological appearance of nerve cells after 45 days. Our results are in line with those of previous reports demonstrating that ETH (3 g/kg/day) through gavage induced moderate hematuria, severe congestion, and moderate infiltration of inflammatory cells in different tissues [5], [33], [38], [54], [61], [66], [67]. Our results indicated that, pathological damages were alleviated by DZM, as a protective agent, in the alcoholic mice. Also, as shown in Fig. 6E, lymphocytes and plasma cell aggregation were observed in the group received ETH at a dose of 2.5 g/kg. Whereas treatment in combination with DZM decreased level of perivascular edema, congestion, and cell swelling significantly in comparison with only administration of ETH at a dose of 2.5 g/kg (Figs. 6F and 6G).

Finally, according to our results, DZM has antioxidant effect when used simultaneously with ETH. Our findings revealed that, combination therapy with DZM and ETH has beneficial effects on oxidative damage induced by ETH. It can be concluded that, antioxidant effect of DZM may be due to its free radicals scavenging properties, but further investigation is needed in order to confirm this hypothesis.

## 5 Conclusion

In conclusion, our results indicated that the subchronic ETH administration induced brain oxidative damage, mitochondrial disruption, and serious damage to the brain cells. Whereas co -treatment of DZM and ETH showed pronounced antioxidant effect. Further, combination therapy improved oxidative damage, mitochondrial function, and pathological changes in brain cells after intoxication by ETH. Thus, ETH in combination with DZM may be a better therapeutic candidate for prevention of brain oxidative damage induced by ETH.

## CRediT authorship contribution statement

Conceived and designed the experiments; **Hamidreza Mohammadi and Seyed Khosro Ghasempouri**. Performed the experiments; **Hamidreza Mohammadi and Zahra Askari**. Analyzed and interpreted the data; **Seyed Khosro Ghasempouri, Zahra Askari Hamidreza Mohammadi**. Contributed reagents, materials, analysis tools or data; **Seyed Khosro Ghasempouri, Zahra Askari Hamidreza Mohammadi**. Wrote the paper; **Seyed Khosro Ghasempouri, Zahra Askari Hamidreza Mohammadi**.

## Declaration of Competing Interest

The authors declare that they have no known competing financial interests or personal relationships that could have appeared to influence the work reported in this paper.

## Data Availability

No data was used for the research described in the article.

## References

[1] Mukherjee S. (2007). Effects of ethanol consumption on different organs – a brief overview. Asian J. Biochem..

[2] Manzo-Avalos S., Saavedra-Molina A. (2010). Cellular and mitochondrial effects of alcohol consumption. Int. J. Environ. Res. Public Health.

[3] Foss J.D. (2018).

[4] Crews F.T., Nixon K. (2008). Mechanisms of neurodegeneration and regeneration in alcoholism. Alcohol. Alcohol..

[5] Das S.K., Vasudevan D. (2007). Alcohol-induced oxidative stress. Life Sci..

[6] Bondy S.C. (1992). Ethanol toxicity and oxidative stress. Toxicol. Lett..

[7] Farooqui A.A. (2014).

[8] McCool B.A. (2011). Ethanol modulation of synaptic plasticity. Neuropharmacology.

[9] Albano E. (2006). Alcohol, oxidative stress and free radical damage. Proc. Nutr. Soc..

[10] Tapia-Rojas C. (2017). Mitochondrial Diseases.

[11] Murphy M.P. (2009). How mitochondria produce reactive oxygen species. Biochem. J..

[12] Tschopp J. (2011). Mitochondria: sovereign of inflammation?. Eur. J. Immunol..

[13] Jung M.E. (2015). Alcohol withdrawal and cerebellar mitochondria. Cerebellum.

[14] Bustamante J. (2012). Alterations of motor performance and brain cortex mitochondrial function during ethanol hangover. Alcohol.

[15] Haorah J., Rump T.J., Xiong H. (2013). Reduction of brain mitochondrial β-oxidation impairs complex I and V in chronic alcohol intake: the underlying mechanism for neurodegeneration. PLOS One.

[16] Lamarche F. (2013). Mitochondrial permeability transition pore inhibitors prevent ethanol-induced neuronal death in mice. Chem. Res. Toxicol..

[17] Sarnowska A. (2009). Diazepam neuroprotection in excitotoxic and oxidative stress involves a mitochondrial mechanism additional to the GABAAR and hypothermic effects. Neurochem. Int..

[18] Aerden L. (2004). Diazepam reduces brain lesion size in a photothrombotic model of focal ischemia in rats. Neurosci. Lett..

[19] Kyung-Hye H., Endo S., Olsen R.W. (1996). Diazepam-insensitive GABA A receptors in rat cerebellum and thalamus. Eur. J. Pharmacol..

[20] DeLorey T., Olsen R. (1992). Gamma-aminobutyric acidA receptor structure and function. J. Biol. Chem..

[21] Tallman J.F. (1980). Benzodizepines: biochemistry to function. Benzodiazepines.

[22] Michler A., Wolff J.R. (1991). GABA accelerates excitotoxic cell death in cortical cultures: protection by blockers of GABA-gated chloride channels. Brain Res..

[23] Cooney G., Heydtmann M., Smith I.D. (2018). Baclofen and the alcohol withdrawal syndrome – a short review. Front. Psychiatry.

[24] Lejoyeux M., Solomon J., Adès J. (1998). Benzodiazepine treatment for alcohol-dependent patients. Alcohol. Alcohol..

[25] Mayo-Smith M.F. (1997). Pharmacological management of alcohol withdrawal: a meta-analysis and evidence-based practice guideline. JAMA.

[26] Muzyk A.J. (2013). The role of diazepam loading for the treatment of alcohol withdrawal syndrome in hospitalized patients. Am. J. Addict..

[27] Bielka K., Kuchyn I., Glumcher F. (2015). Addition of dexmedetomidine to benzodiazepines for patients with alcohol withdrawal syndrome in the intensive care unit: a randomized controlled study. Ann. Intensive Care.

[28] Fathi H. (2015). Oxidative damage induced by retching; antiemetic and neuroprotective role of Sambucus ebulus L. Cell Biol. Toxicol..

[29] Bameri B. (2018). Evidence for the involvement of the dopaminergic system in seizure and oxidative damage induced by tramadol. Int. J. Toxicol..

[30] Zhang F. (2008). In vitro effect of manganese chloride exposure on energy metabolism and oxidative damage of mitochondria isolated from rat brain. Environ. Toxicol. Pharmacol..

[31] Ebrahimzadeh M.A. (2019). Attenuation of brain mitochondria oxidative damage by Albizia julibrissin Durazz: neuroprotective and antiemetic effects. Drug Chem. Toxicol..

[32] Bradford M.M. (1976). A rapid and sensitive method for the quantitation of microgram quantities of protein utilizing the principle of protein-dye binding. Anal. Biochem.

[33] Amrani A. (2017). Alcohol induced hepato cardiotoxicity and oxidative damage in rats: the protective effect of n-butanol extract of green tea (Camellia sinensis (L.) Kuntze). Cardiovasc. Haematol. Disord. -Drug Targets (Former. Curr. Drug Targets-Cardiovasc. Hematol. Disord. ).

[34] Diamond I., Gordon A.S. (1997). Cellular and molecular neuroscience of alcoholism. Physiol. Rev..

[35] Lamarche F. (2003). Acute exposure of cultured neurones to ethanol results in reversible DNA single-strand breaks; whereas chronic exposure causes loss of cell viability. Alcohol. Alcohol..

[36] Genova M.L. (2004). Mitochondrial Pathogenesis.

[37] Sun G.Y., Sun A.Y. (1985). Ethanol and membrane lipids. Alcohol.: Clin. Exp. Res..

[38] Haorah J. (2005). Alcohol‐induced oxidative stress in brain endothelial cells causes blood‐brain barrier dysfunction. J. Leukoc. Biol..

[39] Brooks P. (2000). Brain atrophy and neuronal loss in alcoholism: a role for DNA damage?. Neurochem. Int..

[40] Carrero R.J., Husain K. (2005). Renal oxidative stress in chronic alcohol-induced hypertension. Ethnicity Dis..

[41] BASU S. (2018). Protective role of crude extract of amorphophallus campanulatus against ethanol-induced oxidative renal damage. Asian J. Pharm. Clin. Res.

[42] Dinu D., Nechifor M.T., Movileanu L. (2006). Ethanol‐induced alterations of the antioxidant defense system in rat kidney. J. Biochem. Mol. Toxicol..

[43] Oyenihi O.R. (2016). Hepato-and neuro-protective effects of watermelon juice on acute ethanol-induced oxidative stress in rats. Toxicol. Rep..

[44] Dikranian K. (2005). Ethanol-induced neuroapoptosis in the developing rodent cerebellum and related brain stem structures. Dev. Brain Res..

[45] Green D.R., Kroemer G. (2004). The pathophysiology of mitochondrial cell death. science.

[46] Kitagaki H. (2007). Ethanol‐induced death in yeast exhibits features of apoptosis mediated by mitochondrial fission pathway. FEBS Lett..

[47] Chen G. (2012). Autophagy is a protective response to ethanol neurotoxicity. Autophagy.

[48] Lee Y.-j (2004). Roles of the mammalian mitochondrial fission and fusion mediators Fis1, Drp1, and Opa1 in apoptosis. Mol. Biol. Cell.

[49] Suen D.-F., Norris K.L., Youle R.J. (2008). Mitochondrial dynamics and apoptosis. Genes Dev..

[50] Karbowski M. (2002). Spatial and temporal association of Bax with mitochondrial fission sites, Drp1, and Mfn2 during apoptosis. J. Cell Biol..

[51] Yuan H. (2007). Mitochondrial fission is an upstream and required event for bax foci formation in response to nitric oxide in cortical neurons. Cell Death Differ..

[52] Bonet-Ponce L. (2015). On the mechanism underlying ethanol-induced mitochondrial dynamic disruption and autophagy response. Biochim. Biophys. Acta (BBA)-Mol. Basis Dis..

[53] Farooqui A. (2014).

[54] Hoek J.B., Cahill A., Pastorino J.G. (2002). Alcohol and mitochondria: a dysfunctional relationship. Gastroenterology.

[55] Hatwalne M.S. (2012). Free radical scavengers in anaesthesiology and critical care. Indian J. Anaesth..

[56] Schwartz R.D. (1994). Postischemic diazepam is neuroprotective in the gerbil hippocampus. Brain Res..

[57] Schwartz R.D. (1995). Diazepam, given postischemia, protects selectively vulnerable neurons in the rat hippocampus and striatum. J. Neurosci..

[58] Chelli B. (2004). Peripheral benzodiazepine receptor ligands: mitochondrial transmembrane potential depolarization and apoptosis induction in rat C6 glioma cells. Biochem. Pharmacol..

[59] Guizzetti M. (2010). Ethanol inhibits neuritogenesis induced by astrocyte muscarinic receptors. Glia.

[60] Montoliu C. (1995). Ethanol increases cytochrome P4502E1 and induces oxidative stress in astrocytes. J. Neurochem..

[61] Mukherjee S. (2013). Alcoholism and its effects on the central nervous system. Curr. Neurovascular Res..

[62] Aydin S. (2002). N-acetylcysteine reduced the effect of ethanol on antioxidant system in rat plasma and brain tissue. Tohoku J. Exp. Med..

[63] Calabrese V. (1998). Stress proteins and SH-groups in oxidant-induced cellular injury after chronic ethanol administration in rat. Free Radic. Biol. Med..

[64] Veenman L. (2004). Peripheral-type benzodiazepine receptor density and in vitro tumorigenicity of glioma cell lines. Biochem. Pharmacol..

[65] Wilms H. (2003). Involvement of benzodiazepine receptors in neuroinflammatory and neurodegenerative diseases: evidence from activated microglial cells in vitro. Neurobiol. Dis..

[66] Liu H. (2014). Acute ethanol-induced changes in edema and metabolite concentrations in rat brain. BioMed. Res. Int..

[67] Uysal M. (1989). Ethanol-induced changes in lipid peroxidation and glutathione content in rat brain. Drug Alcohol Depend..

[68] Naseer M.I. (2009). Vitamin-C protects ethanol induced apoptotic neurodegeneration in postnatal rat brain. Pak. J. Med. Sci..

